# Large‐scale cluster randomised trial reveals effectiveness of *Helicobacter pylori* eradication for gastric cancer prevention

**DOI:** 10.1002/ctm2.70229

**Published:** 2025-02-12

**Authors:** Zong‐Chao Liu, Wen‐Qing Li

**Affiliations:** ^1^ State Key Laboratory of Holistic Integrative Management of Gastrointestinal Cancers, Beijing Key Laboratory of Carcinogenesis and Translational Research, Department of Cancer Epidemiology Peking University Cancer Hospital & Institute Haidian District Beijing China

1

Gastric cancer (GC) remains a critical public health concern, ranking among the most prevalent cancers and leading causes of cancer‐related deaths globally.^1^ The burden is particularly pronounced in East Asia, Latin America and Eastern Europe, with nearly half of all new cases and related deaths occurring in China alone. *Helicobacter pylori*, classified as a Group I carcinogen, plays a crucial role in gastric carcinogenesis. *H. pylori* infection in China accounts for approximately 340 000 new cancer cases annually,^2^ contributing to more than 70% of all GC cases nationwide. In 2018, the infection was responsible for 78.5% of non‐cardia GC and 62.1% of cardia GC.^3^ While randomised trials have demonstrated the efficacy of *H. pylori* eradication for GC prevention,^4^ in 2014, the International Agency for Research on Cancer highlighted a lack of data to quantify the overall benefits and risks of implementing large‐scale, population‐based eradication programs in real world.^5^

Recently, the Mass Intervention Trial in Shandong (MITS), China, a community‐based, cluster‐randomised controlled superiority intervention trial, provided new evidence on the effectiveness of population‐wide *H. pylori* intervention in preventing GC (ChiCTR‐TRC‐10000979, https://www.chictr.org.cn).^6^ Conducted across 980 villages in 10 townships in Linqu, Shandong, the trial involved 180 284 participants, divided into three groups based on baseline *H. pylori* infection status. *H. pylori*‐positive individuals were assigned to receive either 10‐day quadruple anti‐*H. pylori* treatment (20 mg omeprazole bid, 750 mg tetracycline tid, 400 mg metronidazole tid and 300 mg bismuth citrate bid, *n* = 52 026) or symptom alleviation treatment (a single dosage of 20 mg omeprazole and 300 mg bismuth citrate, *n* = 50 304), while *H. pylori*‐negative individuals (*n* = 77 954) did not receive any treatment.

The trial's 11.8‐year follow‐up (2011–2022) confirmed a 13% reduction in GC incidence among all treated participants and a 19% reduction among those with successful *H. pylori* eradication, compared to the symptom alleviation group.^6^ The authors also reported a more clinically informative measure: a number needed to treat (NNT) of 141 patients to prevent one GC case across all treated individuals, which was improved to 96 among those who achieved successful eradication.^6^ These NNT estimates from MITS were higher (indicating comparatively modest effect magnitude) than those reported in prior trials and meta‐analyses,^7^ mainly due to the usage of a partially effective symptom alleviation treatment in MITS, which included omeprazole and bismuth rather than a pure placebo. Notably, 15.1% of participants in the symptom alleviation group tested negative for *H. pylori* posttreatment, which may have diminished the relative efficacy of the eradication therapy. The current follow‐up length of this trial can also be another factor influencing the effect estimates, as most other trials reporting lower NNTs were followed up much longer than MITS.^7^ Despite the modest effect size, the findings suggest that over 85 000 new GC cases could be prevented annually in China,^6^ emphasising the trial's real‐world relevance for population‐wide *H. pylori* screen‐and‐treat programs. These make the trial findings more informative for guiding implementation of population‐wide *H. pylori* screen‐and‐treat programs based on real‐world effectiveness rather than only proffering treatment efficacy estimates.

The trial also highlighted the effectiveness of early intervention of *H. pylori* infection, with a 35% reduction in incidence and a 43% reduction in mortality among individuals aged 25–45 years.^6^ These findings support prioritising younger adults for *H. pylori* eradication, particularly in high‐risk regions where the treatment has demonstrated significant economic value. Given the role of *H. pylori* infection in triggering the transition of normal mucosa to non‐atrophic gastritis and the markedly long latency of gastric lesions progressing to cancer, screening and treatment for the infection in early adulthood would be desirable for interrupting progression of the precancerous cascade towards GC, thus achieving the maximal effect for GC prevention. It should also be noted that effective *H. pylori* screen‐and‐treat strategies will have additional benefits in reducing other important clinical conditions, including peptic ulcer disease, dyspepsia, iron deficiency and other non‐gastrointestinal conditions.^4^

Enhancing eradication outcomes is important for efficient prevention of GC. In the MITS trial, bismuth quadruple therapy with tetracycline and metronidazole achieved a 72.9% eradication success rate given a combined antibiotic resistance rate of 5.32% in a pilot study, underscoring the importance of addressing antibiotic resistance to prevent eradication failure. Antibiotic resistance is a leading cause of *H. pylori* treatment failure and currently poses a significant challenge in China's healthcare system. A nationwide survey in China reported resistance rates exceeding 50.8% for clarithromycin in individuals aged 40–60 years, as well as 47.2% resistance to levofloxacin with higher prevalence among women.^8^ To counteract rising resistance rates, current international guidelines recommend antibiotic susceptibility testing before initiating treatment or after the first treatment failure.^6^ For implementing population‐wide *H. pylori* intervention strategies, it is also essential to consider geographic variations in resistance, which may be influenced by area‐specific factors such as socioeconomic conditions, hygiene practices, healthcare access and patterns of antibiotic use.^8^

The MITS demonstrated the feasibility of *H. pylori* eradication in outpatient or community settings, with manageable side effects and minimal risks of severe adverse events. While secondary outcomes, such as overall mortality and the incidence of other cancers, did not show significant changes, extended follow‐up may uncover additional benefits. This is particularly relevant for cardia GC, for which the association with *H. pylori* is strongest in Asia. Current data suggest that *H. pylori* contributes to 40.7%–62.1% of cardia GC in China,^3^, ^9^ underscoring the need for ongoing research to fully elucidate the role of *H. pylori* eradication in preventing cardia GC.

While the study supports the general feasibility of population‐level intervention for GC prevention, the authors caution against a ‘one‐size‐fits‐all’ approach during implementation. From a practical perspective, family‐based *H. pylori* screening and treatment may further reduce the infection recurrence rate compared to a single‐patient approach, though well‐designed, large‐scale randomised trials are needed to provide robust evidence.^10^ Future research efforts are advocated towards establishing a comprehensive system to refine prevention strategies, targeting high‐risk individuals and beneficial subgroups. To this end, the biorepository established during the MITS trial offers a valuable resource for biomarker discovery and molecular studies.^5^ Extended follow‐up of this large‐scale cohort would further refine prevention strategies by leveraging this biorepository in combination with advanced multi‐omics techniques to unravel the complexities of gastric carcinogenesis and address *H. pylori* treatment heterogeneity in GC prevention.

## AUTHOR CONTRIBUTIONS

Wen‐Qing Li conceived the study and contributed to the study design. Zong‐Chao Liu and Wen‐Qing Li contributed to data analysis, interpretation, writing and edited the manuscript.

## FUNDING INFORMATION

This study was funded by the Noncommunicable Chronic Diseases‐National Science and Technology Major Project (2023ZD0501400‐2023ZD0501402), Beijing Hospitals Authority's Ascent Plan (DFL20241102) and Science Foundation of Peking University Cancer Hospital (2022‐27).

## ETHICS STATEMENT

The original trial and study follow‐up were approved by the institutional review boards of Peking University Cancer Hospital & Institute (approval no. 20090724) and Technical University of Munich (approval no. 55718 S‐KK), and all participants provided written informed consent.
